# Ultrawide electrical tuning of light matter interaction in a high electron mobility transistor structure

**DOI:** 10.1038/srep16812

**Published:** 2015-11-18

**Authors:** Shovon Pal, Hanond Nong, Sergej Markmann, Nadezhda Kukharchyk, Sascha R. Valentin, Sven Scholz, Arne Ludwig, Claudia Bock, Ulrich Kunze, Andreas D. Wieck, Nathan Jukam

**Affiliations:** 1 Lehrstuhl für Angewandte Festkörperphysik, Ruhr-Universität Bochum, D-44780 Bochum, Germany; 2 AG Terahertz Spektroskopie und Technologie, Ruhr-Universität Bochum, D-44780 Bochum, Germany; 3 Lehrstuhl für Werkstoffe und Nanoelektronik, Ruhr-Universität Bochum, D-44780 Bochum, Germany

## Abstract

The interaction between intersubband resonances (ISRs) and metamaterial microcavities constitutes a strongly coupled system where new resonances form that depend on the coupling strength. Here we present experimental evidence of strong coupling between the cavity resonance of a terahertz metamaterial and the ISR in a high electron mobility transistor (HEMT) structure. The device is electrically switched from an uncoupled to a strongly coupled regime by tuning the ISR with epitaxially grown transparent gate. The asymmetric potential in the HEMT structure enables ultrawide electrical tuning of ISR, which is an order of magnitude higher as compared to an equivalent square well. For a single heterojunction with a triangular confinement, we achieve an avoided splitting of 0.52 THz, which is a significant fraction of the bare intersubband resonance at 2 THz.

Ultrastrong light-matter interaction is one of the key aspects of quantum photonics and has been a subject of great interest for superconducting qubits^1^,^2^, atomic^3^,^4^,^5^ and quantum dot^6^ systems. In addition to electronic and optical frequencies, strongly coupled light-matter systems can also be obtained for quantum well intersubband transitions coupled to mid-infrared^7^ and THz^8^,^9^ resonators. When a transition is strongly coupled to a cavity resonance, the bare frequencies of the uncoupled system shift to new frequencies. The frequency shift depends on the strength of the coupling and can be explained in terms of periodic exchange of energy through vacuum Rabi oscillations^10^,^11^ or coupling between the oscillators in a strongly dispersive system^12^. Since the frequency shift is proportional to the light-matter interaction, switchable and tunable strong coupling is of interest for filters and modulators.

Artificial structures of sub-wavelength sizes with novel electromagnetic properties, commonly known as metamaterials (MMs)^13^,^14^,^15^, can be used to create resonators to study light-matter interactions. Recently, ultrastrong coupling experiments have been performed, where the cyclotron resonance of the two dimensional electron gas (2DEG) is coupled to THz MM resonators^16^,^17^,^18^,^19^,^20^. The transitions between the Landau levels are brought into resonance with the MMs by tuning the magnetic field. However, electrical tunability is more preferable for devices. In 2012, Gabbay *et al.*^21^ modeled the coupling of MMs to electrically tunable ISRs in a square well for mid-infrared frequencies. These devices are later demonstrated experimentally by Benz *et al.*^22^,^23^,^24^. In order to excite the intersubband transitions, the incident electric field should have a component parallel to the growth direction. For an inductor-capacitor (LC) MM resonator^14^,^25^,^26^, the electric field in the capacitor region is perpendicular to the growth direction. However, due to local bending of the electric field on the capacitor edges, the MM mode can be strongly coupled to ISRs in multiple quantum wells^27^,^28^,^29^,^30^. High electron mobility transistor (HEMT) structures, on the other hand, form the building blocks of most modern high-speed electronic circuits. Monolithic integration of MMs with HEMT permits amplitude modulation of THz radiation at MM resonances up to a few MHz^31^ in the linear light-matter regime. Such integrated structures not only show potential application towards electrically tunable THz devices, but also in the ultrastrong light-matter interaction regime.

In this contribution, we demonstrate strong coupling of ISR in a single heterojunction formed in a HEMT structure to a THz MM. This is achieved by electrically tuning the ISR with a complementary doped epitaxial gate^32^. The asymmetric potential at the heterojunction enables wide tuning of the ISRs in comparison to a square well. The low vacuum band-offset of the complementary epitaxial gate further enhances the electrical tunability. In addition, the triangular well has greater oscillator strength. This allows the ultrastrong light-matter interaction regime to occur between a single heterojunction with a triangular confinement and a MM resonator.

## Results

### Designing the Metamaterial-HEMT Device

**Figure 1:**
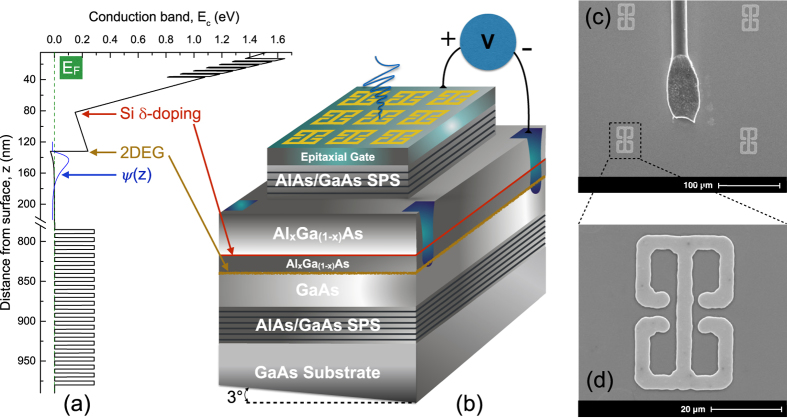
Device design. (**a**) Conduction band edge, E_*c*_, of our device when a certain bias is applied at the gate such that the ground state is below the Fermi energy. The ground wavefunction is also plotted within the well. (**b**) Schematic layer sequence of the device and the corresponding electric field direction of the incident infrared radiation, which couples to the resonator. The bottom surface of the device is wedged at an angle of 3° in order to avoid the Fabry Pérot fringes in transmission measurements. SEM micrograph showing (**c**) metamaterials and wire bonding, (**d**) metamaterial unit cell.

Intersubband transitions in semiconductor quantum well can be designed to cover the wide infrared region of the electromagnetic spectrum^33^,^34^. The device is based on intersubband transition in 2DEG, formed in a GaAs/Al_*x*_Ga_1−*x*_As heterojunction of a HEMT structure, Fig. 1(a). The HEMT consists of a heterostructure that is delta-doped in the Al_*x*_Ga_1−*x*_As layer, 50 nm from the heterojunction. The electrons from the delta-doped donors diffuse to the GaAs layer. This results in the presence of a strong built-in space charge fields between the carriers in the GaAs and donors in the Al_*x*_Ga_1−*x*_As. The potential near the heterojunction can be approximated to a triangular potential with a constant slope in GaAs layer and a vertical barrier from the GaAs/Al_*x*_Ga_1−*x*_As conduction band-offset. The electronic motion perpendicular to the surface is quantized, resulting in the formation of quasi-two-dimensional-subbands. By varying the external bias, the slope of the triangular potential along with the transition energies can be tuned. In contrast to a square well with abrupt barriers on either side, a triangular well with gradual slope (on the GaAs side) will have a much greater effect on tuning the subband spacings by electrical bias. The external bias is applied by making contacts to the 2DEG and a heavily doped p-type region on the surface, which forms an epitaxial gate. The complementary doping of the gate leads to a low vacuum band-offset (typically 0.5 eV), thus a lower depletion of carriers and hence a wide voltage tunability of the device (or intersubband energies). The thickness of the epitaxial gate is only 20 nm, which is much less than the skin depth. This makes the epitaxial gate transparent to THz radiation. Further details on the layer sequence of the sample are described in the methods section.

Arrays of double-sided Au split-ring resonators (SRR) are deposited on top of the device, shown in Fig. 1(b,c). The SRRs have sub-wavelength sizes and are designed to have a cavity resonance comparable to the first intersubband transition in the 2DEG. The resonators are similar to electrical-inductor-capacitor (ELC) circuit, where the inductive regions correspond to the loops of the SRRs and the capacitive regions correspond to the gaps in the loops of the SRRs. As shown in Fig. 1(d), the double-sided SRRs consists of two single SRRs that are mirror images of each other for reflections along the central metal line^16^. The current travels in a clockwise direction for one of the single SRRs and in anticlockwise for the other. Thus the induced magnetic fields from the two SRRs will average to zero and the double SRR will only couple to external electric fields through the capacitive regions.

**Figure 2:**
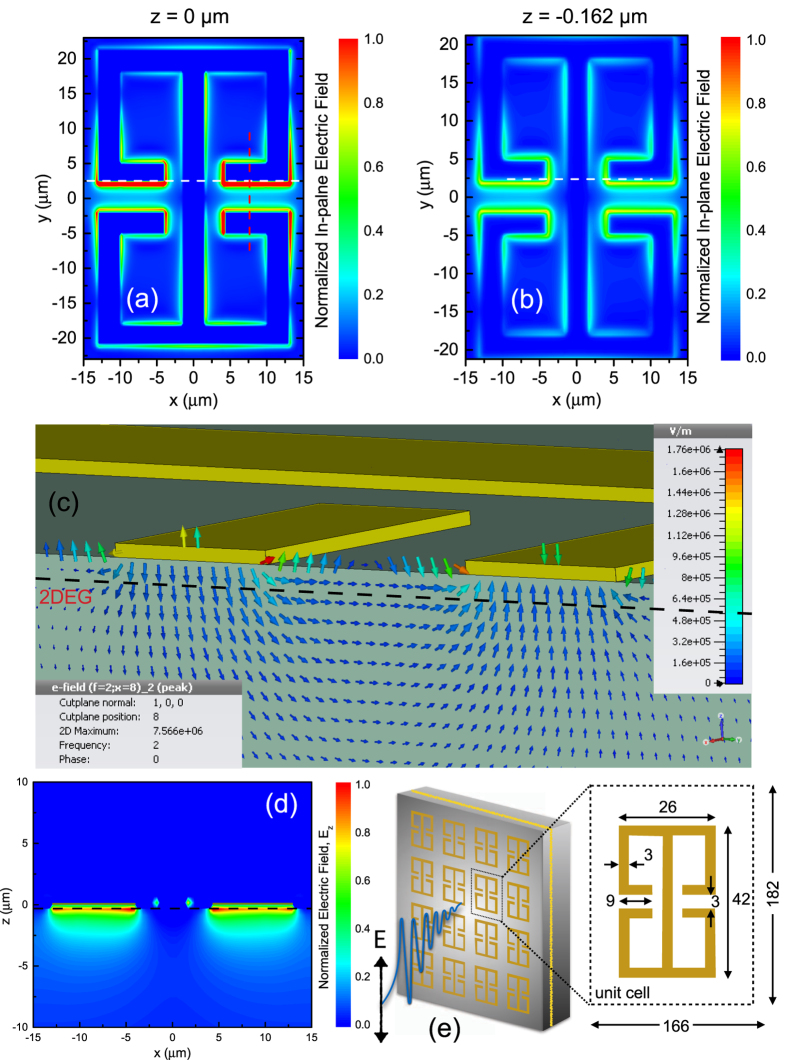
FDTD simulations of metamaterials. In-plane electric field distribution, 
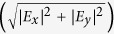
, at (**a**) *z* = 0 *μ*m (on top of the surface) and (**b**) *z* = −0.162 *μ*m (in the 2DEG layer, 162 nm below the surface). (**c**) Snapshot of the fringing electric fields along the vertical cut shown by the red-dashed line in (**a**). (**d**) The field distribution, 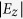, in the growth direction along the cut shown by the white lines in (**a**,**b**). The black-dashed line in (**c**,**d**) represents the position of the 2DEG. (**e**) A schematic representation of the metamaterial structure and the electric field component of the polarized excitation source for the TDS measurements. All dimensions are in units of *μ*m. The metamaterials are arranged in a unit cell of 166 *μ*m × 182 *μ*m. All electromagnetic field simulations are performed at 2 THz.

Finite difference time domain (FDTD) simulations using a commercial software package (CST microwave studio) is used to calculate the electric field distribution and the frequency response of the MM modes. The simulations are carried out for a single unit cell with periodic boundary condition in *x*−*y* plane and open boundaries (with spaces) in *z* plane. The electric field direction of the incident signal is polarized along *y* direction. A schematic of the arrangement used for electromagnetic simulation along with the dimensions of MM are shown in Fig. 2(e). The in-plane field distribution 
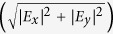
 is strongly enhanced over sub-wavelength volumes around the capacitive regions of the double-sided SRRs, Fig. 2(a,b). Intersubband transitions only couple to electric fields polarized in the growth direction perpendicular to the surface. Although the fields between the capacitor sections are predominantly in-plane, there is a significant out-of plane field from fringing effects (see Fig. 2(c)) on the surface^35^,^36^. Figure 2(d) shows the electric field distribution, 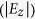 in the growth direction along the cut, indicated by the white line in Fig. 2(a,b). All field simulations are performed at 2 THz. This frequency corresponds to the intersubband transition from the ground to the first excited state in the heterojunction of the HEMT structure. The equivalent electrostatic capacitance is an important parameter of the microcavities, which play a key role in strong light-matter coupling. This is due to the fact that the Rabi frequency is directly proportional to the equivalent capacitance^37^. The longer capacitive arm (9 *μ*m, see Fig. 2(e)) of the MMs used ensures larger coverage of the heterojunction, which enables more electrons to participate in the coupling. Moreover, it is also observed that the electric filed in the z-direction is significantly enhanced. While the coupling strength has only a very weak dependence on the actual geometry of the MM design^38^, the number the quantum well in the structure play a significant role. Since a single heterojunction (with a triangular confinement potential) is employed in our integrated device, a greater coverage and strong electric fields in the growth direction become essential, which are met by the use of current MM design.

#### 2DEG Intersubband Resonances

**Figure 3:**
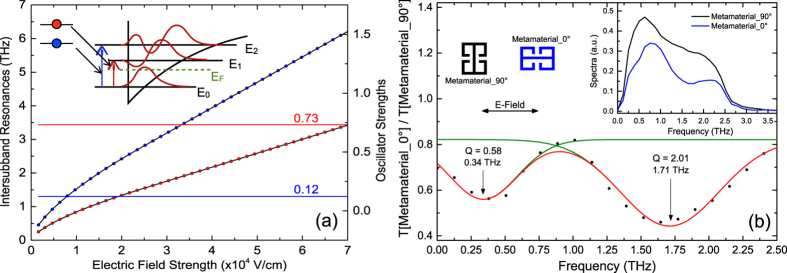
1D Poisson simulation and characterization of metamaterials. **(a)** Dependence of the intersubband resonances and the oscillator strengths for two intersubband transitions, 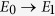 and 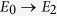, on the electric field strength and hence the bias applied on the gate. The oscillator strengths do not vary with the gate voltage and are fixed for each transition, which is a characteristic feature of the triangular potential well. Inset: Band-schematic of the first three subbands in a 2DEG with respect to the Fermi level at an intermediate gate voltage where only the ground subband is below the Fermi level. **(b)** The transmission of the incident polarized electric field for the horizontal orientation of the metamaterial (Metamaterial_0°) normalized to the transmission for the vertical orientation of the metamaterial (Metamaterial_90°). The green spectra are results of deconvolution and the red curve is the reconstructed transmission spectrum. Inset: Spectra corresponding to the two orientations of the metamaterials. The schematic shows the orientations of the meta-structures with respect to the electric field direction in the THz TDS measurements.

In order to characterize the energy eigenvalues of the 2DEG confined in a triangular potential well, a self-consistent Schrödinger-Poisson equation is solved using the 1D Poisson solver^39^. The solution is carried out for different bias applied to the gate. The dependence of the intersubband spacings of two resonances, 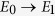 and 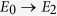 are plotted as a function of the electric field strength in Fig. 3(a), where *E*_0_, *E*_1_ and *E*_2_ corresponds to the energies of ground, first and second subbands respectively. By tuning the gate voltage, the confinement potential and hence the electric field strength, *F* can be altered. Under the triangular well approximation^40^, the shift of the ISR to higher energies is given by^41^:


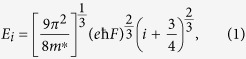


where *i* (0, 1, 2, …) represent the indices of the subbands, m^*^ is the effective mass of electrons in GaAs and *e* is the electronic charge. The normalized oscillator strengths, *f*_*ij*_, are calculated from the transition matrix elements, 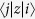, corresponding to the ISRs by using the following relation:


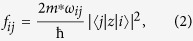


where 
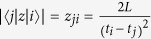
, with 
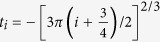
41. The quantity, *L* is defined as the electric length, given by 
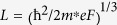
. The normalized oscillator strength for 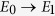 transition is calculated to be 0.73 while that of 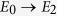 transition is 0.12. According to the sum rule, the normalized oscillator strengths of all transitions sum up to 1, which indicates transition to higher levels are very weak. Under the framework of triangular well approximation, the oscillator strengths are calculated to be independent of the electric field strength (Fig. 3(a)). This is unique compared to that of the square potential well as shown by Benz *et al.*^23^, where there is a small contribution of the quantum-confined Stark shift^42^ to the oscillator strengths in the presence of external bias. Due to asymmetric confinement, the ISRs can be tuned with a magnitude one order higher as compared to an equivalent square well. For a triangular well with an effective thickness of 20 nm, if the electric field is tuned by 4.5 × 10^4^ V/cm, the ISR (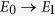) can be tuned by 35 meV, while for an equivalent square well for the same change in electric field the ISR is tuned only by 3 meV. The experimentally observed ISRs are blue-shifted due to resonant screening from higher subbands, also known as the depolarization effect^40^,^41^,^43^ and is explained in the methods section.

### Characterization of metamaterials

The MMs are characterized by THz time-domain spectroscopy (TDS) at room temperature under purged conditions, to avoid the water absorption lines. The experimental details of the set-up are explained separately in the methods section. At room temperature the thermal energy, *k*_*B*_*T* (=25 meV), is greater than the subband energy spacings. This result in the occupation of higher subbands, which consequently prevents us from observing the ISR (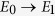) from the 2DEG. Hence under this condition, the response from the sample is purely due to MMs. The transmission is measured for two orientations of the MMs, Fig. 3(b). In the inset, the corresponding spectra are shown. The electric field of the THz radiation couples to the MMs only when they are oriented parallel to the optical table, marked as Metamaterial_0° in Fig. 3(b). The normalized transmission is plotted by dividing the transmitted intensity with respect to the MM orientation when the fields do not couple. Two strong resonances are observed in the transmission spectrum at 0.34 THz and 1.71 THz. The quality factors of the resonance frequencies at 0.34 THz and 1.71 THz are found to be 0.58 and 2.01 respectively. These resonances have different origin described as follows: The lower frequency at 0.34 THz is due to the LC equivalent circuit where the current circulates in the inductive part and the field is concentrated between the capacitor plates. The resonance at 1.71 THz is due to the half wave resonance^44^ arising from the sides of MM. There are additional losses from the epitaxial layer, damping and change in the effective dielectric constant of the layers with the change in temperature. From the quality factor of the resonance at 1.71 THz, we estimated the losses to be around 0.85 THz. On cooling the sample, the cavity resonance will be blue shifted according to^45^: 
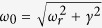
, where *ω*_0_ is the bare cavity resonance without any losses, *ω*_*r*_ is the resonance of the MMs including losses and *γ* is proportional to the combined effect of ohmic losses in the metal and radiative damping in the cavity. The resulting resonance frequency without losses is calculated to be 1.9 THz, which is very close to the observed resonance at 4.2 K.

### Charging spectroscopy of the 2DEG

**Figure 4:**
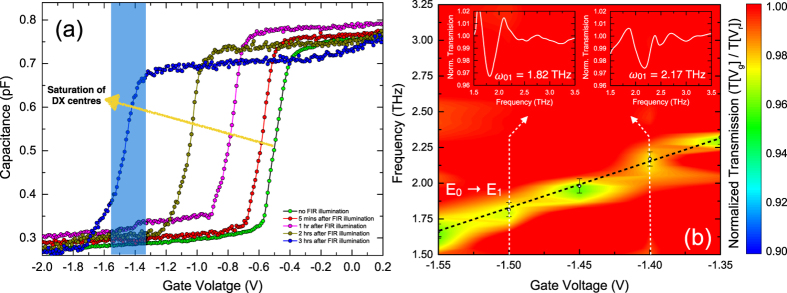
Capacitance-Voltage Spectroscopy and Intersubband Resonance. (**a**) Dependence of capacitance on the voltage applied to the gate. With illumination, the visible part of the spectrometer radiation source saturates the DX centres and hence the threshold voltage shifts to more negative values. When a quasi-stable condition is reached, and the threshold does not shift any further, all donors are saturated. The blue shaded region highlights the range of voltage over which the FTIR transmission spectra are taken. (**b**) Contour plot of the normalized transmission spectra corresponding to the bare intersubband transition (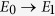), without metamaterials. The black-dashed line is a guide to the eye, which shows the shift of the transition to higher energies with the increase of the bias. Inset: Normalized transmission spectra corresponding to two voltage points; *V*_*g*_ = −1.5 V (left inset) and *V*_*g*_ = −1.4 V (right inset), as indicated by the white-dashed lines.

The charging of the 2DEG subbands is observed by capacitance-voltage spectroscopy at 4.2 K, shown in Fig. 4(a). The change in capacitance between the top gate and the bottom 2DEG layer is measured as a function of the gate voltage by applying DC + AC voltage to the gate and measuring the AC component of the current across the ohmic. The steep increase of capacitance in the voltage ranging from −0.6 V to −0.4 V indicates that the 2DEG layer is filled with charge carriers (green curve). When the sample is illuminated with far-infrared (FIR) broad-band source (Hg-arc lamp), the threshold voltage shifts more to negative values. This is due to the activation of the donor-exchange (DX) centres^46^. As the sample is illuminated longer, more donor atoms from the *δ*-doped AlGaAs layer ionize and less bias is required to charge the 2DEG subbands, resulting in the shift of charging slope to more negative biases. When the charging spectrum does not shift any more (shown by the blue curve), a steady state is reached. The activation of the DX-centres is a result of residual near band-gap illumination (after filtering by black polyethylene window) from the FIR source. The blue shaded region in Fig. 4(a) marks the voltage range where density-chopped infrared transmission measurements are performed. The Schrödinger-Poisson equations are solved over the shaded voltage range, corresponding to the electric field strengths plotted in Fig. 3(a).

### Strong coupling of metamaterials with 2DEG

**Figure 5:**
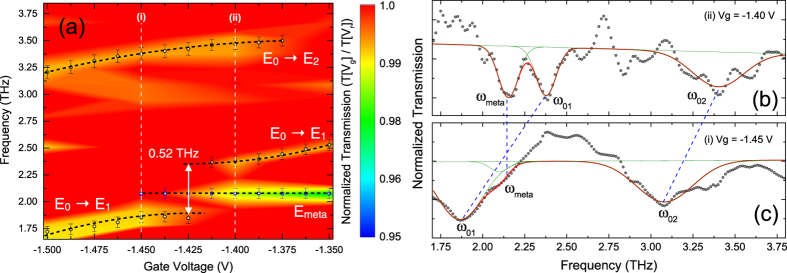
Observation of strong coupling. (**a**) Strong coupling is observed in the transmission spectra by tuning the gate voltages for the 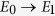 transition with the metamaterial resonance at around 2 THz, while no coupling is observed for the other intersubband transition, 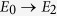. The coupling of the cavity resonance with the 2DEG intersubband resonance is seen as a split at the cross over point between the two resonances with a split-gap of 0.52 THz. The black-dashed lines are guide to the eye. The normalized transmission spectra at two voltage points, (i) *V*_*g*_ = −1.45 V and (ii) *V*_*g*_ = −1.40 V are shown in (**b**,**c**) respectively. Each spectral deconvolution shows three dips (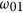, 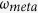 and 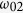) corresponding to the points shown in (**a**). The blue dashed lines show the shift of the respective transitions.

An FTIR transmission set-up with a rapid scan BRUKER IFS113V interferometer is used for the density chopped infrared transmission spectroscopy to observe the strong coupling of the MM cavity resonance with the 2DEG ISRs. As observed in the charging spectrum, at a bias of −2 V, the conduction band of the 2DEG is pulled above the Fermi level and hence completely depleted of carriers. This is used as the reference voltage in the chopping-scheme at which sample transmission is recorded. The gate voltage is then increased to −1.55 V, where only one subband is below the Fermi level and corresponding transmission measurements are performed. The voltages are changed alternatively and the respective spectra are co-added and averaged over time. This is repeated for several gate voltages, keeping the reference voltage same. All normalized transmission spectra are collectively plotted in a contour-plot. At first, the bare intersubband resonance is characterized in a sample, which does not have the MM layer on top. This is shown in Fig. 4(b). In order to access the intersubband transitions, the sample is tilted at an angle to obey the polarization selection rule. On increasing the gate bias from −1.55 V to −1.35 V, the transition frequency from the ground subband to the first excited subband increases from 1.6 THz to 2.5 THz. It can be seen that tuning the gate voltage by 200 mV, the intersubband transition is shifted by nearly 1 THz. The possibility of wide electrical tuning results from the asymmetry in the triangular confinement potential at the GaAs-AlGaAs heterojunction. For the sample with MMs on top, it is observed that at low temperature, the cavity resonance shifted to 2.12 THz due to lower losses as compared to the room temperature measurements. This is a favorable situation since the cavity resonance now lies within the tuning range of the intersubband transitions. Fig. 5(a) shows the contour plot of the normalized transmission spectra for the integrated device. The Q-factor of the MM resonance has dramatically improved to a value of 16.9. At *V*_*g*_ = −1.425 V, a clear splitting of the ISR can be observed when the ISR (*ω*_01_) of the 2DEG crosses the resonance of the MMs at 2.12 THz. The width of the splitting is found to be 0.52 THz. Two normalized transmission spectra at *V*_*g*_ = −1.45 V and *V*_*g*_ = −1.40 V, shown by the white line in Fig. 5(a) are plotted in Fig. 5(b,c). The spectra are smoothed in order to carry out the spectral deconvolution. Three peaks, marked as *ω*_01_, *ω*_*meta*_ and *ω*_02_ can be seen corresponding to the points marked in Fig. 5(a). As described before, the ISRs are blue shifted by 1.2 meV as compared to theoretical values plotted in Fig. 3(a), due to the resonant screening from the higher subbands. It is observed that at *V*_*g*_ = −2 V, not only the 2DEG channel gets completely depleted of charge carriers but also the response from the capacitive element of the MMs is screened due to charges in the epitaxial layer. By applying bias on the gate below the MM, a significant modulation of the intensity (or amplitude) of the cavity resonance is observed^31^,^47^. Thus in our chopping scheme, the MM resonance at 2.12 THz disappears for biases lower than −1.45 V and begins to appear for biases higher than −1.425 V. In our experiments, we limit the bias applied on the gate to −1.3 V. Since with increasing bias the slope of the triangular confinement becomes steeper, the second subband is pulled below the Fermi level, which makes the transition scheme different, which is not desirable.

## Discussion

The 2DEG and MMs in our device can be considered as two oscillators, one of which has a fixed frequency and the frequency of the other oscillator (2DEG) is tuned by the gate voltage. When the frequencies of both the oscillators are similar, they form a coupled system and an anti-crossing phenomenon is observed. This results in a periodic transfer of energy between the 2DEG and the microcavity through vacuum Rabi oscillations, which is proportional to the splitting at the anti-crossing point. Using the common oscillator model, described by Gabbay *et al.*^21^, the strength of the coupling is found to be directly proportional to the strength of the splitting, described in the methods section. The quantity, Ω, defined as the coupling strength is given by:


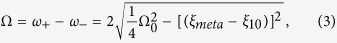


where Ω_0_ is the bare coupling strength, *ξ*_*meta*_ and *ξ*_10_ represent the losses in the metamaterials and ISRs and is proportional to the linewidths of the respective transitions. The coupling strength in our experiment is found to be 0.26 THz, while the bare coupling strength is calculated to be 0.28 THz. There is no significant change in the two coupling strengths. The ratio of the splitting to the sum of full-width at half maximum of both the resonances, 
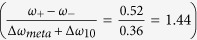
 is found to be greater than one. The normalized coupling ratio 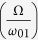 in our experiment is found to be 0.13, which is a significant fraction of the intersubband transition energy. Interestingly, in our experiment the bare cavity resonance is present in the range of voltages where the coupling regime lies and beyond. The presence of the uncoupled resonator signal at the anticrossing point has been reported before in atom-cavity system^48^ and its solid-state analogues such as quantum dot-cavity systems^6^,^49^. While in the atom-cavity system this is a result of the fluctuation in the number of atoms in the cavity, in case of dot-cavity systems it is due to the fluctuation of the emitter energy over time. Following a similar chain of arguments, our system is a solid-state analogue where the quantum well replaces the quantum dot and the metamaterials replace the photonic crystal^6^ or Fabry Pérot cavity^49^. The fluctuation in the emitter energy results from the fluctuation of the gate voltage since the frequency of the intersubband transitions depends very sensitively on the applied gate voltage and hence on the carriers. This results in the observation of the third peak from the cavity in the anticrossing point (see 5(a)), when the two systems are strongly coupled. For higher voltage (above −1.375 V) regime, the presence of cavity mode is simply due to the fact that the two oscillators in our device are no longer coupled and the two resonances appear independently; while the intersubband transition keeps shifting to higher energies (due to quantum confined Stark effect), the cavity resonance is fixed. It is to be noted that a difference between the spectra at two different gate biases is measured. Thus a stronger MM cavity mode represents a change in the absorption strength of the MMs with change of bias. Besides, the presence of charges with increased bias will also change the quality factor of the cavity. Due to the use of a single heterojunction, the low contrast ratio hinders the simultaneous observation of both polaritonic modes at the same gate bias. A double high electron mobility transistor structure, one inverted with respect to the other can be envisaged. With top- and back-gates, the structure can be tuned from either side, which would enhance the contrast of the resonances observed. Of course care should be taken to design the structure identically with the same ground to first excited state transition energies.

In conclusion, we show strong coupling of the 2DEG ISR in a heterostructure with a triangular confining potential to the cavity resonance of a THz MM by driving the device from an uncoupled state to a strongly coupled state via electrical tuning of ISR. Due to asymmetric confinement potential, the tuning of the ISR is found to be one order of magnitude higher in comparison to an identical square well. The use of epitaxial gate proved to be advantageous for the use of direct MMs and consequently tune the ISR at the same time. With a proper chopping scheme, we successfully demonstrated that the measurement could be performed in one integrated sample without the need of additional reference sample. This is one of the remarkable features of our device. Moreover, we succeeded to achieve ultrastrong light-matter interaction by employing a single heterojunction of a high electron mobility transistor structure. The vacuum Rabi frequency is 13% of the bare intersubband resonance frequency, which is a high value if we consider that this is achieved only by electrical tuning of the intersubband transitions in a single heterojunction with an asymmetric triangular confinement potential.

## Methods

### Sample Design

The sample is grown on a semi-insulating GaAs (100) substrate by molecular beam epitaxy. At first, 20 periods of AlAs/GaAs (5 nm/5 nm) short period superlattice (SPS) are grown to trap segregating impurities at the interfaces and to smoothen the surface for growth. A 650 nm thick GaAs layer is grown, followed by a 50 nm Al_0.34_Ga_0.66_As spacer layer. This is followed by a Si-*δ* doping and then a 45 nm Al_0.34_Ga_0.66_As layer. Another 6 periods of SPS layer comprising of 1 nm GaAs and 3 nm AlAs are grown, capped by a 13 nm GaAs layer above which the epitaxial gate^32^ is grown. The epitaxial gate has a 15 nm thick carbon-doped GaAs layer and 30 periods of carbon-*δ* doped and 0.5 nm carbon-doped GaAs layers, which lead to an effective carrier density of N_*A*_ = 2 × 10^19^ cm^−3^. To establish a contact with the 2DEG layer, 162 nm below the surface, the corners of a 5 mm × 6 mm sample are first etched down by 100 nm (Fig. 1(b)) and then indium is diffused in a reaction chamber in an inert atmosphere of argon and nitrogen. The sample is then mounted on a chip carrier and wires are bonded by wedge-bonding. The structures are processed on the epitaxial gate, shown schematically in Fig. 1(b), by conventional UV-photolithography, evaporation of Cr/Au (10/200 nm) and lift-off technique. Scanning electron micrographs (SEM) of the MMs and a wire bonding are shown in Fig. 1(c,d).

### THz time domain spectroscopy

A Ti:Sa laser with 80 fs pulse duration (centre wavelength of 800 nm) and a repetition rate of 80 MHz is used to generate THz radiation by exciting an inter-digitated photoconductive antenna processed on a GaAs substrate at an applied DC bias. The experiment is performed in transmission geometry with a NIR-power of 200 mW on the antenna. The detection is based on electro-optic sampling of the THz electric field by employing a birefringent, 2 mm thick ZnTe crystal. The electric field component of the source is in a direction parallel to the optical table. Two 90° off-axis parabolic mirrors are used to collimate and focus the THz beam on the sample, which is fixed on a rotation mount. Another two parabolic mirrors are used to collect the signal from the sample and focus it on the ZnTe crystal. The transmission measurements are performed to observe the response of the metamaterials alone in the device under purged conditions.

### Oscillator Strengths and Depolarization Shifts

Under the triangular well approximation, the intersubband energy edges, *E*_*i*_, are directly proportional to *F*^2/3^, where *F* is the electric field strength. Now the oscillator strength for the transition between two subbands in a triangular potential well is proportional to the square of the matrix elements, which in turn is proportional to the square of the electric length, *L*. The electric length is inversely proportional to *F*^1/3^. This implies that the oscillator strength does not depend on the field strength in a triangular well and is a constant given by:


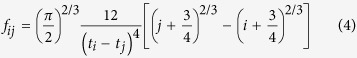


where, 
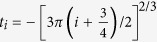
41. For *i* = 0 and *j* = 1, *f*_01_ = 0.73 and for *i* = 0 and *j* = 2, *f*_02_ = 0.12. Taking in to account the allowed transitions to higher subbands (*j* = 3, 4, …), the total oscillator strength, 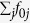, sums up to 1. The normalized energy eigenfunctions for the ground and the first excited intersubbands under triangular confinement, are given by^41^:


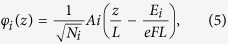


where, *N*_*i*_ are the normalization constants given by *Ai*′(*t*_*i*_) and 
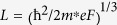
. Including the resonant screening effects, the intersubband energies are blue shifted by


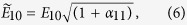


where *α*_11_ is the depolarization factor, which according to Ando^50^ is given by 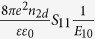, where *n*_2*d*_ is the 2D carrier density; *ε* is the dielectric constant of GaAs; *ε*_0_ is the permittivity of free space. 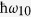 is the energy spacing corresponding to the transition, 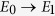 and the overlap factor, *S*_11_ has the dimensions of length and is given by^50^:


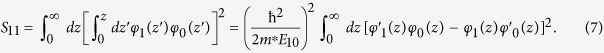


The carrier density is however not a fixed value and changes with the change in bias. Besides the bias also changes the slope of the confinement thus changing the overlap-integral (*S*_11_). At a bias of −1.4 V, corresponding to a carrier density of 2 × 10^11^ cm^−2^, the depolarization shift is calculated to be 1.2 meV. The influence of resonant screening becomes important when the level spacings are smaller and the carrier densities are high.

### Coupling Strength

The system is assumed to be comprised of two oscillators that are tuned from uncoupled to strongly coupled regime by tuning the ISR of the 2DEG with an external gate bias, making use of quantum-confined Stark effect. Following the model of coupled oscillators, as developed by Gabbay *et al.,*^21^ the coupling between the two resonators can be described by a simple 2 × 2 matrix:


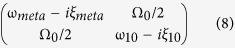


where *ω*_10_, *ω*_*meta*_ are the resonances and *ξ*_10_, *ξ*_*meta*_ are the damping factors corresponding to ISR and metamaterials; Ω_0_ is the bare coupling strength of the oscillators. The matrix can be diagonalized to get the upper and lower eigenvalues of the coupled system as:


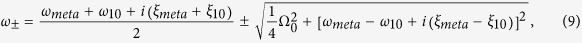


Subtracting the frequencies, we obtain:


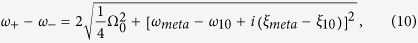


At the anticrossing point, both the resonators start oscillating with equal and real frequencies, the coupling strength, Ω, can be expressed as:


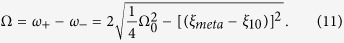


From the two (upper and lower) branches of the transitions, the coupling strength, Ω, is found to be 0.26 THz. The damping factors corresponding to the resonances are *ξ*_*meta*_ = 0.13 THz and *ξ*_10_ = 0.23 THz. The bare coupling strength, Ω_0_, is calculated to be 0.28 THz which similar to the coupling strength.

## Additional Information

**How to cite this article**: Pal, S. *et al.* Ultrawide electrical tuning of light matter interaction in a high electron mobility transistor structure. *Sci. Rep.* **5**, 16812; doi: 10.1038/srep16812 (2015).
